# Ultrasound-Responsive Antibacterial Surfaces on Titanium Alloys: A Narrative Review

**DOI:** 10.3390/jfb17090450

**Published:** 2026-09-06

**Authors:** Jiayang Gao, Yang Liu, Chenxin Zhan, Meixi Wu, Xinman Wang, Yongheng Zhu, Yuqin Qiao

**Affiliations:** 1 International Research Center for Food & Health, College of Food Science and Technology, Shanghai Ocean University, Shanghai 201306, China; 2334202@st.shou.edu.cn; 2 Materdicine Lab, School of Life Sciences, Shanghai University, Shanghai 200444, China

**Keywords:** ultrasound-responsive, antibacterial surfaces, titanium alloys, orthopedic implants

## Abstract

Ultrasound-responsive antibacterial therapies represent a cutting-edge approach to combating implant-associated infections, particularly advantageous because of ultrasound’s non-invasive nature, deep penetration, and ability to target infected areas. However, most previous studies have been devoted to ultrasound-responsive nanomaterials, hydrogels, and polymer systems. Research on ultrasound-responsive surfaces on titanium remains in its early stages and is extremely limited. This review primarily focuses on the design principles and construction strategies for ultrasound-responsive antibacterial surfaces on titanium-based implants, key factors affecting acoustic energy conversion efficiency, and the approaches to enhance antibacterial efficiencies. Based on acoustic energy conversion mechanisms and the primary factors responsible for bacterial killing, this review classifies antibacterial surfaces into sonothermal surfaces, sonodynamic surfaces, and multimodal combined surfaces. Moreover, this review discusses perspectives and challenges regarding biocompatibility, long-term antibacterial activity after implantation, and future clinical translation.

## 1. Introduction

Implant-associated infection (IAI) remains a significant challenge in orthopedic surgery, which can result in severe consequences for personal health and implant longevity. Clinically, these infections necessitate combination antibiotic therapy or additional surgical interventions to achieve effective eradication [1,2,3]. However, antimicrobial resistance significantly threatens global public health by compromising the efficacy of conventional antibiotics. According to an earlier study, there were approximately 5 million deaths associated with antimicrobial resistance in 2019 [4]. Titanium (Ti) and its alloys are the leading options for orthopedic implants due to exceptional biocompatibility and mechanical properties [5]. However, titanium-based implant surfaces are susceptible to bacterial adhesion, colonization, and ultimately biofilm formation, which involves complex microbial communities encased in extracellular polymeric substances [6]. Biofilms generally display increased resistance to both host immune defenses and antibiotics and are exceptionally difficult to eradicate. Subsequently, biofilms can further disrupt immune balance, create a niche that facilitates bacterial persistence, and provoke chronic inflammation through the release of toxins and inflammatory mediators. The ongoing infections can progressively destroy peri-implant bone, increase the risk of implant failure, and significantly diminish a patient’s quality of life [7,8,9]. In recent years, multiple strategies have been developed to endow titanium with antibacterial activities, encompassing the incorporation of inorganic and organic antibacterial agents, the design of mechano-bactericidal nanostructures, and the construction of local antibiotic delivery systems [10,11,12]. However, these strategies have certain limitations restricting their clinical translation. For example, current antibacterial agents often release continuously once implanted, which can be exhausted when chronic infections develop after more than 1 month. Moreover, protein adsorption on implant surfaces can reduce the antimicrobial effectiveness of nanostructure-induced mechano-bactericidal activity [13].

To overcome these barriers, intelligent external-field-stimulated antibacterial strategies have emerged as a promising and targeted therapeutic modality with improved biosafety and numerous advantages [14,15,16]. For example, on-demand bactericidal activity triggered by a therapeutic stimulus could avoid excessive adverse effects on biological tissues induced by uncontrolled release of antibacterial agents. Moreover, different therapeutic stimuli possess distinct tissue penetration depths, giving them unique advantages in combating bacterial infections at various sites. Among these therapeutic stimuli, such as light, heat, electricity, and mechanical forces, ultrasound offers the advantage of low cost, ease of operation, noninvasiveness, and deeper tissue penetration (1~10 cm), especially for bone and large joints [17,18,19]. The development of ultrasound-responsive antibacterial strategies has primarily focused on nanomaterials, yet various types of nanomaterials have been shown to induce oxidative stress and to raise biosafety concerns. Thus, constructing in situ ultrasound-responsive antibacterial surfaces or coatings on implants represents a promising solution. In contrast, fabricating ultrasound-responsive coatings faces several issues. First, nanomaterials have exceptionally high specific surface areas and many surface defect sites, whereas coatings exposed to physiological fluids have relatively limited surface features, resulting in poor sonocatalytic performance [20]. Second, acoustic waves generally reflect and refract at the liquid/solid interface rather than penetrating the coating, which significantly reduces the effective acoustic energy received [21]. Third, a higher adhesion strength between the coating and the substrate is required because dislodgement of the coating or its components during ultrasound exposure may cause inflammation and biosafety concerns. Owing to these factors, research on ultrasound-responsive surfaces on titanium remains in its early stages and is extremely limited [22].

**Figure 1:**
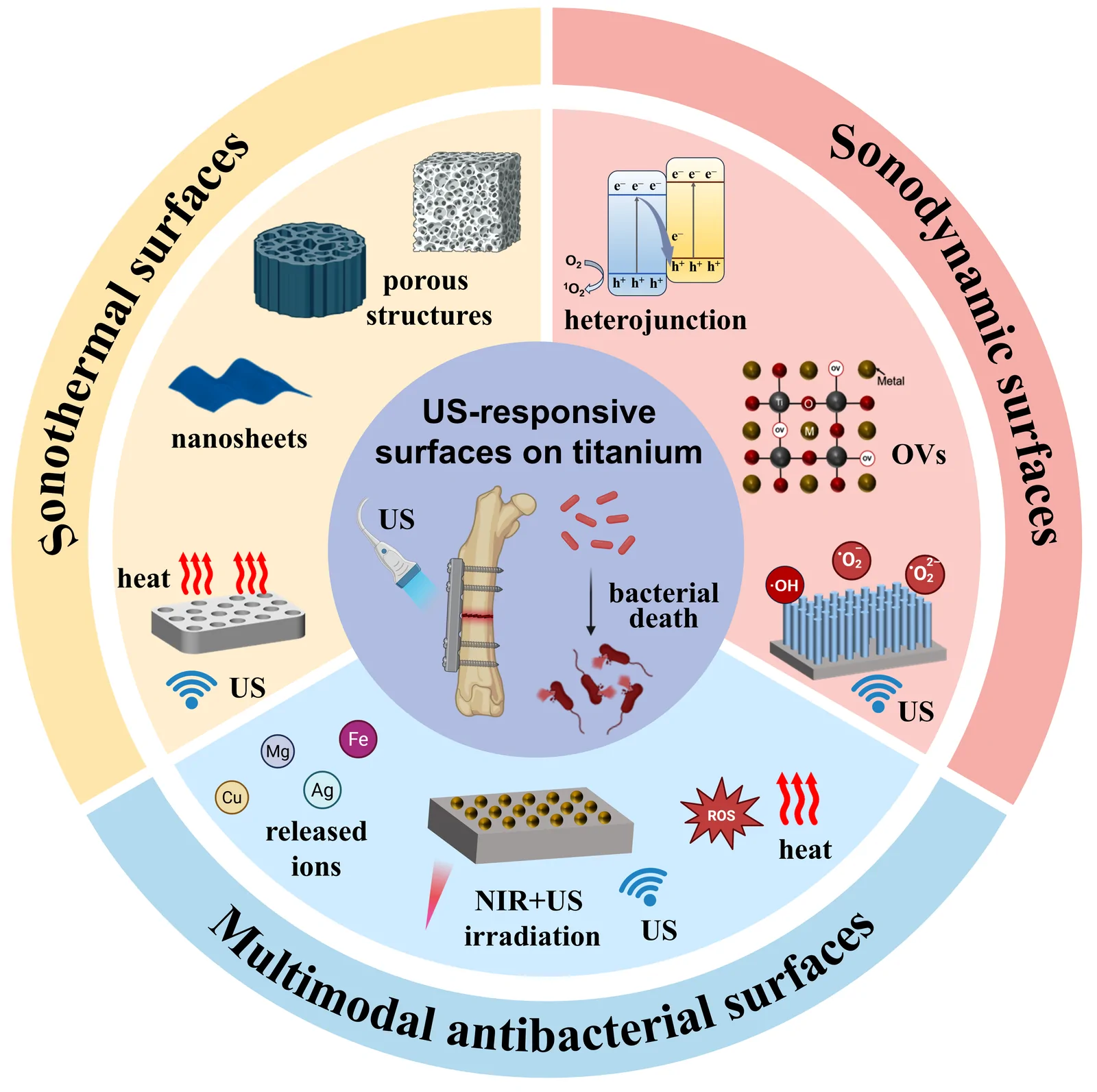
Schematic illustration of ultrasound-responsive antibacterial surfaces on titanium alloys. Illustrations were created in BioRender. Qiao, Y. (2026) https://BioRender.com/wjus8a6.

Over the past few years, although numerous reviews on ultrasound-responsive antibacterial therapy have been published from multiple perspectives, including material types, potential applications, and construction approaches, no dedicated review specifically covers ultrasound-responsive antibacterial surfaces on titanium-based implants [23,24]. In this review, we focused on research progress and the underlying mechanisms of ultrasound-driven antibacterial surfaces for titanium-based implants over the past five years (Figure 1). Based on how acoustic energy is mainly converted and the primary bacterial factors involved, we classify these surfaces into three categories: sonothermal surfaces, sonodynamic surfaces, and multimodal combined surfaces. Beyond antibacterial performance, other biological properties relevant to implant biological responses, including osteogenesis, angiogenesis, and immunomodulation, will also be addressed in this review. Finally, the present challenges and future perspectives regarding biosafety and clinical translation are discussed. This review provides deeper insight into the design principles and critical factors that determine the antibacterial efficiency of ultrasound-responsive surfaces on titanium-based implants, and offers guidance on achieving an optimal balance between combating bacterial infection and the biosafety required for clinical translation (A search strategy is given in Appendix A).

## 2. Sonothermal Surfaces

Sonothermal therapy (STT) is a newly emerging approach for treating implant-associated infections [25,26]. When interacting with the ultrasound-responsive nanomaterials, the deeply penetrating ultrasound can induce cavitation, convert acoustic energy to heat, and cause locally increased temperature [27,28,29]. The thermal effect not only disrupts the cell membrane but also breaks down the protective matrix around the film via combined mechanical damage, ultimately resulting in greater bactericidal activity. Meanwhile, the sonothermal effect has been employed in drug-loaded systems to control drug release, which may further enhance antibacterial activities or facilitate other biological functions, such as osteogenesis, angiogenesis, and immune responses. In the past, nanomaterials have been extensively studied and exploited as efficient sound-absorbing materials because of their nanoscale dimensions, large specific surface area, and tunable porosity [30,31]. However, the development of sonothermal surfaces or coatings for orthopedic implants has still been challenging.

Constructing a heterojunction provides a promising strategy to enhance electron motion, generate a built-in electric field at the interface, and achieve sonothermal effects. In a recent study, the authors developed a uniform film composed of metal-red phosphorus (RP) nanoparticles on titanium (Ti-RP) by chemical vapor deposition (CVD) technology [25]. Red phosphorus, an emerging non-metallic photocatalyst and sonosensitizer, has been shown to be nontoxic and can be degraded into biocompatible phosphate and phosphonate after long-term existence in the human body [32,33]. It can be found that, under ultrasound excitation, the electron motion or phonons in the RP and Ti heterojunction could contribute to mechanical-to-thermal transformation. This, combined with acoustic-to-thermal conversion, leads to an obviously improved sonothermal ability, with a temperature elevation of more than 20 °C (from 29.30 °C to 49.70 °C) compared to Ti. The in vitro antibacterial rate of Ti-RP against multidrug-resistant *Staphylococcus aureus* (*MRSA*) increased to 67.52% from 19.7% (Ti) under ultrasound. To further improve the antibacterial efficiency, nitric oxide/nanomesoporous silicon dioxide (SNO) was coated on the surface of Ti-RP (Ti-RP-SNO). Under ultrasound irradiation, Nitric oxide (NO) was released in a controlled manner via cleavage of the heat-sensitive S-N bond. The functional surface, alongside local heat, demonstrated high eradication of *MRSA* (93.40% inhibition rate) using a bone infection model in rats. Furthermore, the H&E staining images indicated that the heat generated by ultrasound irradiation did not induce serious damage to biological tissues.

Ultrasonic metamaterials also enable new possibilities for manipulating acoustic waves based on artificial structures and show promising applications in biomedical imaging and diagnostic systems [34,35,36]. However, the development of acoustic metamaterials has been rarely discussed in biomedical applications such as bone tissue regeneration and implant-associated infections [37,38]. Recently, Guan et al. constructed a metastructure TiO_2_ porous coating on titanium implants by micro-arc oxidation [39]. They first reported that the “honeycomb-like” acoustic metastructure facilitates efficient acoustic-to-thermal conversion by the “acoustic confinement effect”. Then the heat will continue to diffuse outward along the structural skeleton, leading to a higher temperature inside the pore than outside, which can effectively surpass the non-specific heating of conventional coatings. Under ultrasound excitation, the metastructure coating can capture bacteria within its pores and exhibit ideal antibacterial activities (99.69% for *S. aureus*; 99.58% for *E. coli*) through local heat.

As shown above, sonothermal antibacterial surfaces offer many advantages for treating bacterial infections, especially broad-spectrum bactericidal activity without inducing antibiotic resistance. According to previously cited reports, a coating’s sonothermal mechanism may involve ultrasound-activated electron and phonon transport within the heterogeneous interface, as well as increases in local temperature induced by acoustic cavitation [25,39]. It has been reported that acoustic cavitation can break up and remove biofilm from a surface [40]; thus, it inevitably contributes to sonothermal antibacterial therapy. According to Guan’s research, comparative experiments across groups show that cavitation is not the dominant factor in combating bacterial infection [39].

Despite its significant advantages, the clinical translation of ultrasound-responsive surfaces on titanium-based implants faces several challenges. The primary safety concern is that antibacterial efficiency depends heavily on elevated temperatures; that is, higher temperatures increase antibacterial efficiency and shorten bacterial killing time. According to previous studies, localized temperatures reach at least 50 °C after a few minutes of ultrasound exposure and can rise to 50~60 °C by varying ultrasound irradiation parameters. However, most mammalian cells appear to have a critical thermal tolerance threshold (43 °C), and elevated temperature induces significant necrosis and programmed cell death [41]. Therefore, achieving localized hyperthermia with selective bactericidal activity while minimizing damage to surrounding mammalian cells and tissues remains a key challenge. From this perspective, metamaterials offer a new approach to eliminating bacteria entrapped in pores through elevated temperatures.

## 3. Sonodynamic Surfaces

**Figure 2:**
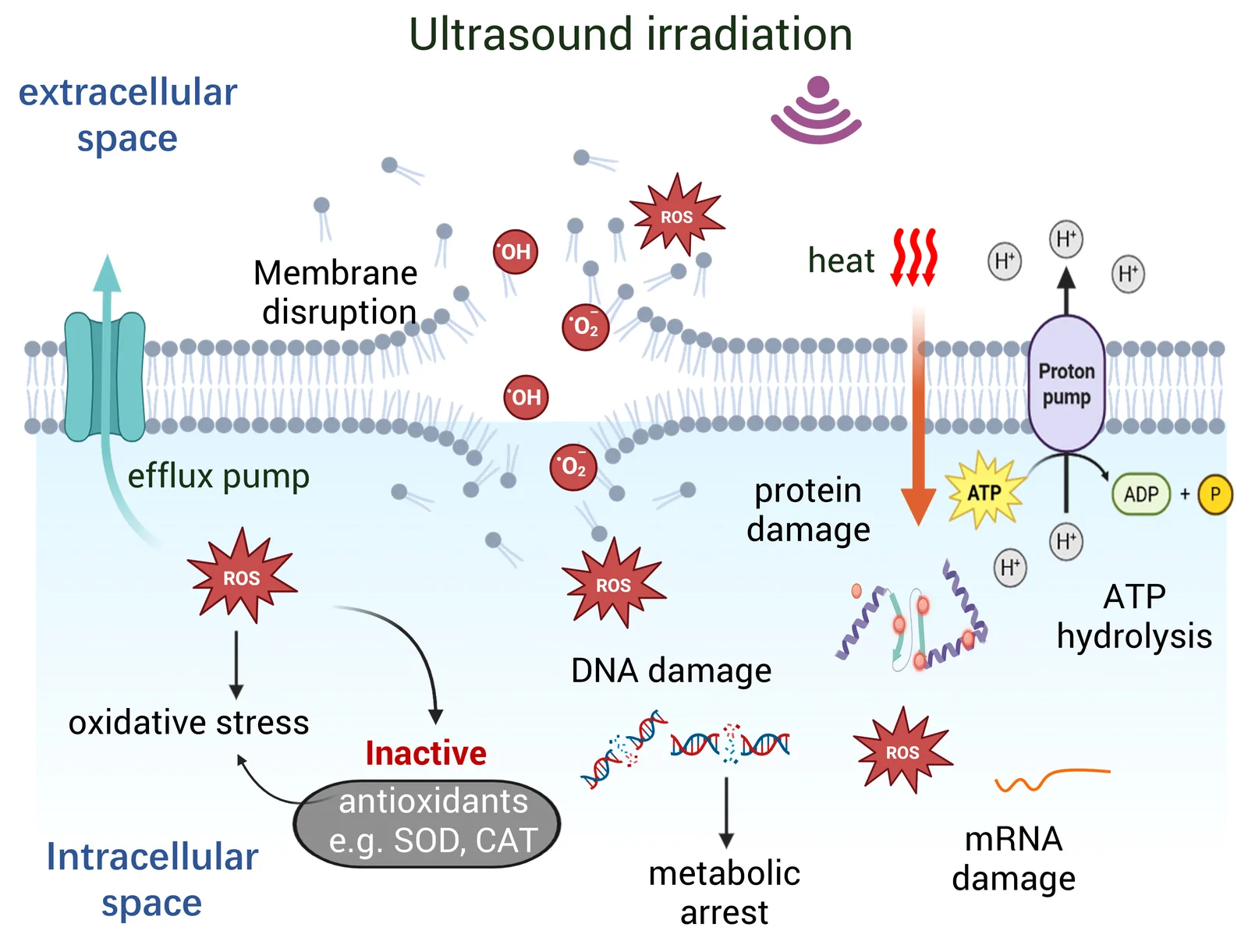
Antibacterial mechanism of sonothermal antibacterial surfaces and sonodynamic surfaces. (Illustrations were created in BioRender. Qiao, Y. (2026) https://BioRender.com/wjus8a6).

Sonodynamic therapy (SDT) is an evolving therapeutic approach that uses ultrasound to activate sonosensitizers, generating reactive oxygen species (ROS), and offering tremendous potential for preventing and combating bacterial infections [42,43,44]. Upon ultrasound excitation, sonosensitizers can absorb acoustic energy, trigger the separation of electron-hole pairs, and drive redox reactions to produce ROS. Excessive ROS formation induces oxidative stress, lipid peroxidation, membrane disruption, and DNA damage, ultimately leading to bacterial cell death (Figure 2) [45,46]. Although titanium dioxide (TiO_2_) based nanomaterials have been widely used as efficient and safe inorganic sonosensitizers in the biomedical field for cancer and bacterial infection treatments [47,48,49], the ROS generation efficiency of pristine TiO_2_ was constrained by its inherent wide band gap (~3.2 eV) and rapid recombination of electron-hole pairs.

It has been reported that doping various metals, such as gold (Au) and copper (Cu), into TiO_2_ coating can narrow down its band gap [50,51]. In a previous study, Li and co-workers demonstrated that the deposition of Au NPs onto TiO_2_ nanotubes (Au-TNT) caused the band gap to decrease from 3.59 eV (TiO_2_ nanotubes, TNT) to 3.50 eV [52]. When irradiated by ultrasound, electrons flow from the TNT to the Au NPs, effectively preventing electron-hole recombination. This can greatly enhance ROS yield, and the antibacterial rates of *P. gingivalis*, *F. nucleatum*, and *S. sanguinis* biofilms in the Au-TNT + US group were 96.59%, 96.25%, and 95.31%, respectively, compared with the Ti group. In another study, Wang et al. constructed an acoustic metastructure Cu-doped defective titanium oxide coating (Cu-TiO_x_) by micro-arc oxidation and plasma immersion ion implantation techniques followed by thermal reduction treatment [53]. The results showed that implanted Cu atoms were redistributed during thermal reduction treatment, leading to lattice distortions that produced band tail states and facilitated the separation of electron-hole pairs. This can effectively promote ROS generation and has shown more than 90% inhibition against both *S. aureus* and *E. coli* under ultrasound irradiation.

Notably, defect engineering of inorganic nanosensitizers has been proven to be a simple yet effective strategy for tuning the band gap to suppress electron-hole recombination, thereby boosting ROS generation [54,55,56]. In a recent study, Chen and colleagues prepared a nanorod-like hydroxyapatite (HA) layer on a TiO_2_ coating by micro-arc oxidation and hydrothermal reaction, and then introduced oxygen vacancies (OVs) and a Mg-O-containing amorphous nanolayer via a one-step thermal-reduction method [57]. The results showed that prolonged thermal reduction led to a higher concentration of oxygen vacancies in the sample, which consequently narrowed the TiO_2_ band gap, enhanced H_2_O adsorption, and improved ROS yield under ultrasound irradiation. This OVs-enriched TiO_2_ coating, combined with a weakly alkaline microenvironment created by the degraded Mg-O layer, endows the functional coating with the highest bactericidal activity against methicillin-resistant *Staphylococcus aureus* (*MRSA*, 99.23%) and *Staphylococcus aureus* (*S. aureus*, 99.87%), as well as a biofilm eradication rate of 70.12% against *MRSA* biofilm.

**Figure 3:**
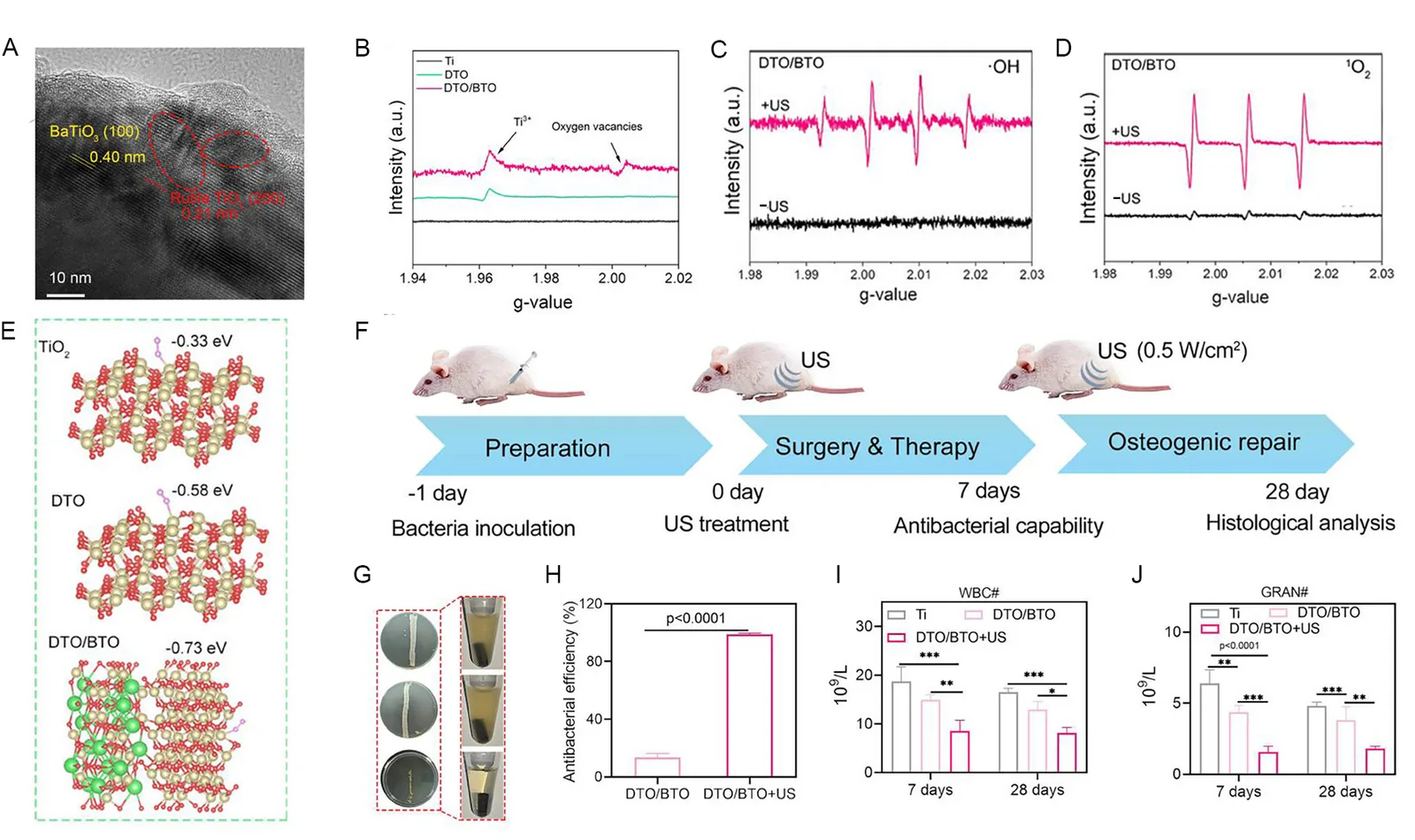
(**A**) HRTEM of DTO/BTO. (**B**) EPR spectra of DTO and DTO/BTO. (**C**,**D**) ESR spectra of ·OH and ^1^O_2_. (**E**) Oxygen adsorption location and oxygen adsorption energy of different samples. (**F**) A diagram of the animal experimental design. (**G**) In vivo antibacterial ability of bacteria-infected samples after implantation for 7 days. (**H**) The corresponding antibacterial efficiency of bacteria extracted from the infected implants was assessed after incubation for 24 h. (**I**) WBC and (**J**) gran counts from blood from the different groups tested at 7 and 28 days post-surgery. Statistical significance was denoted as * *p* < 0.05, ** *p* < 0.01, *** *p* < 0.001. WBC and GRAN are abbreviations of leukocytes and neutrophil granulocytes. (Adapted from Ref. [61]).

Although introducing OVs promotes the generation and separation of electron-hole pairs, ROS generation and sonocatalytic performance remain unsatisfactory because of the rapid recombination [58,59]. In this regard, piezoelectric nanomaterials and heterojunctions have been employed as key coating components to enhance the electron-hole separation [24,60]. A range of inorganic piezoelectric materials have been reported, such as BaTiO_3_, ZnO, and ZnS. Recently, a defective TiO_2_ and barium titanate (BaTiO_3_) heterojunction enriched with Ti^3+^ species was fabricated on Ti using a laser fusion technique under high-temperature and rapid-cooling conditions (DTO/BTO, Figure 3) [61]. As shown in Figure 3B , the existence of OVs was characterized by electron paramagnetic resonance (EPR) spectroscopy. The results showed that OVs narrowed the band gap of TiO_2_, enabling better electron generation under ultrasound activation, and that the asymmetric spin states in DTO/BTO accelerated ultrasonic charge transfer and inhibited the complexation of electron-hole pairs, improving ROS yield for bacterial scavenging. Comparatively, the antimicrobial rate of DTO/BTO against *S. aureus* reached 99.83%, whereas that of TiO_2_ was 8.36%. Similarly, a hydroxyapatite/ BaTiO_3_ composite coating was developed by loading tetragonal BaTiO_3_ nanoparticles on a uniform array of HA nanorods (Ti-HA/BTO), and nitrogen-doped coatings (Ti-HA/N-BTO) can be obtained using urea as a reducing agent and a nitrogen precursor [62]. The results showed that interstitial nitrogen doping can induce lattice distortion and a band gap in BaTiO_3_, enhancing its piezoelectric performance and ultrasonic catalytic activity. For the Ti-HA/BTO group, the antibacterial rates against *S. aureus* and *MRSA* reached 57.57 ± 2.61% and 55.44 ± 2.97%, respectively. After nitrogen doping, the antibacterial rates increased significantly and reached 98.15 ± 0.64% (*S. aureus*) and 98.76 ± 0.22% (*MRSA*). In another study, an ultrasound-responsive array of nanorod-like core–shell heterojunctions was prepared on a Ti substrate [63]. Then OVs and residual compressive stresses were introduced into TiO_2_ shells and ZnO cores (p-ZnO/TiO_2__−__x_), respectively, via annealing in argon and ice cooling. Under ultrasound, OVs stimulated the separation of electron-hole pairs and further minimized electron-hole recombination via the ZnO-generated piezoelectric field due to residual compressive stress. The array p-ZnO/TiO_2−x_ exhibits high bacteria-killing (~99.87%) and biofilm-eradicating efficacy (~80%) against *MRSA*. Based on the above discussion, combining defect and heterojunction modifications can yield notably enhanced sonodynamic activity compared with a single-modification strategy.

In addition to piezoelectric nanomaterials, recent studies have also shown that traditionally considered centrosymmetric semiconductors such as SrTiO_3_ can exhibit piezoelectricity and pyroelectricity when diffused by OVs or when forming a Schottky junction with a metal [64,65]. For instance, Pan et al. reported that Al doping into SrTiO_3_/TiO_2_ heterojunctions (Al-STNT) can induce oxygen vacancies and lattice distortion of SrTiO_3_, which greatly enhances the generation of ROS [66]. Under ultrasound irradiation, the bactericidal ratios for *P. gingivalis* and *F. nucleatum* grown on STNT (SrTiO_3_/TiO_2_) are less than 50%, but they increase significantly to 80.4% and 82.1% for those bacteria grown on Al-STNT, respectively. Moreover, the Al-STNT endows dental implants with osteogenic activity through Sr ion release. Zhou and co-workers developed a heterojunction coating composed of Zr-doped SrTiO_3_ and Hap (SrTiZrO_3_/Hap) [67]. Under ultrasound, single-atom Zr doping may lead to interfacial polarization, which, combined with electron polarization from the heterointerface’s built-in electric field, enabled piezoelectric catalysis. Under ultrasound exposure, SrTiZrO_3_/Hap achieved 99.3% and 99.7% antibacterial rates against *S. aureus* and *E. coli*, respectively. Meanwhile, SrTiZrO_3_/Hap can promote osteogenic differentiation by activating the PI3K/Akt pathway through its piezoelectric, needle-like surface topography and the release of functional ions, thereby accelerating bone repair.

Ongoing efforts have also explored other effective or microenvironment-responsive inorganic sonosensitizers. For instance, Dai et al. fabricated a CaTiO_3_ coating on titanium via hydrothermal reaction [68]. Under ultrasound stimulation, the CaTiO_3_ coating can produce ·OH radicals to kill *S. aureus*, with the antibacterial ratio reaching 89.66%. After being processed using a corona polarization device, the polarized CaTiO_3_ coating not only retained its excellent sonodynamic activity but also generated electrical stimulation (ES) to promote anti-inflammatory M2-type macrophage polarization and bone formation. In one study, Ji and co-workers functionalized ZnS: Ag quantum dots (ZnS: Ag QDs) with tyramine-modified hyaluronic acid (HA-Tyr) and horseradish peroxidase (HRP) to form a hydrogen peroxide (H_2_O_2_) sensitive composite sonosensitizer (HHTZ), which was then loaded onto a titanium oxide nanotube array (TN-HHTZ) [69]. The results showed that, under ultrasound exposure, Ag doping enhanced the energy conversion efficiency of ZnS, increasing ROS generation. Simultaneously, ultrasound induced the rapid release of HHTZ, which subsequently reacted with H_2_O_2_ in the microenvironment to form a gel. This prolonged the local accumulation of ZnS: Ag QDs and also contributed to boosted sonocatalytic activity.

Despite significant advancements achieved, current sonodynamic antibacterial surfaces face several challenges (summarized in Table 1). On the one hand, antibacterial efficiency may be less effective than expected due to varied pathological conditions of an individual. It has been shown that ROS generation is highly dependent on the local oxygen concentration. Insufficient oxygen resources in the infected microenvironment may limit ROS production and further diminish treatment effectiveness [70,71]. In addition, the release of sonosensitizers may compromise the efficiency of SDT in treating chronic infection [63,69]. Thus, overcoming oxygen dependence to improve ROS generation efficiency and achieving long-term antibacterial activity in infected microenvironments remain urgent issues closely tied to clinical outcomes. On the other hand, ROS play significant roles in cell metabolism, including maintenance of redox homeostasis, regulation of signaling pathways, and stimulation of immune defense [72]. Excessive ROS accumulation triggered by ultrasound may disrupt the balance between ROS and antioxidants, leading to oxidative stress and further damage to mammalian cells [73]. A key challenge is that while high levels of ROS promote robust bactericidal activity, they also damage mammalian cells. Therefore, a systematic investigation into the biocompatibility of ROS at varying concentrations and exposure durations is critically important. Further efforts should aim to achieve the optimal balance between bactericidal activity and biocompatibility.

**Table 1:** Sonodynamic antibacterial surfaces on Titanium alloys.

Ultrasound-Responsive Surfaces	Antibacterial Efficiency	Ultrasound Condition	Ref.
Au@TiO_2_ heterojunction	96.59% (*P. gingivalis* biofilm)	1.5 W/cm^2^, 5 min	[52]
OVs-enriched TiO_2_/BaTiO_3_	99.83% (*S. aureus*)	1.5 W/cm^2^, 20 min	[61]
HA/N-doped BaTiO_3_	98.76% (*MRSA*)	1.5 W/cm^2^, 15 min	[62]
ZnO-TiO_2−x_ heterojunction	99.87% (*MRSA*)	2.5 W/cm^2^, 10 min	[63]
Al-SrTiO_3_/TiO_2_ heterojunctions	82.1% (*F. nucleatum*)	1.5 W/cm^2^, 5 min	[66]
Zr-doped SrTiO_3_/HA	99.3% (*S. aureus*)	1.0 W/cm^2^, 10 min	[67]
CaTiO_3_	89.66% (*S. aureus*)	1.5 W/cm^2^, 10 min	[68]
ZnS: Ag QDs-TiO_2_ nanotube	99.81% (*E. coli*); 97.76% (*S. aureus*)	1.0 W/cm^2^, 10 min	[69]
Au@SrTiO_3_ nanorods array	>90% (*S. aureus* and *E. coli*)	1.0 W/cm^2^, 20 min	[74]
Ti@ZnO nanorods array	99.9% (*S. aureus*)	1.0 W/cm^2^, 5 min	[75]
Au NPs coated BTO (PiezoTi)	90.5% (*S. aureus*)	1.0 W/cm^2^, 10 min	[76]

## 4. Multimodal Antibacterial Surfaces

As discussed above, single-mode SDT or STT has difficulty in completely eradicating bacteria due to insufficient ROS production and temperature elevation under ultrasound [77,78]. To overcome these barriers, combination therapies are under development to boost the antibacterial efficiency (summarized in Table 2) [70,79,80].

Integration of multifunctional additives into surfaces plays a pivotal role in achieving multimodal combination therapies [81,82,83]. Two-dimensional black phosphorus (BP) has been reported to have high near-infrared photothermal conversion (PTT) efficiency and single-oxygen radical (^1^O_2_) generation efficiency [84]. Moreover, BP is also an emerging sonosensitizer, although fast electron recombination limits the sonocatalytic activity. Zeng et al. incorporated black phosphorus nanosheets (BPNSs) and polydopamine (PDA) on titanium (Ti/PDA/BP) [85]. Under NIR irradiation for 10 min, the maximum surface temperatures of Ti, Ti/PDA, and Ti/PDA/BP reached 36.5 °C, 42.9 °C, and 48.4 °C, respectively. Despite relatively higher photothermal effects, the antibacterial rate of Ti/PDA/BP was less than 50% against *S. aureus*. After ultrasound exposure for 20 min, the antibacterial efficiency of Ti/PDA/BP increased to 70.1% against *S. aureus* by inducing a large amount of ·OH radicals. It can be concluded that PTT or SDT alone may be insufficient. Thus, after combined NIR irradiation and ultrasound stimulation, the corresponding antibacterial efficiency increased to 96.6%. In a similar study, Ti samples were treated in an argon (Ar_2_) and sulfur (S) atmosphere to obtain an oxygen-deficient S-doped TiO_2_ surface (Ti-S-TiO_2−x_) [86]. Compared with samples treated in a single Ar_2_ atmosphere (Ti-R-TiO_2−x_), sulfur not only stimulates the creation of oxygen vacancies but also further enhances electron-hole separation efficiency, contributing to significant improvements in both photothermal ability and sonodynamic performance. Recently, Sun et al. fabricated a one-dimensional oxygen-deficient BaTiO_3_ nanorod array on Ti (BaTiO_3−x_) [87]. The surface showed an antibacterial efficiency of 91.15% against *MRSA* due to the generated superoxide radical (·O_2−_) under ultrasound irradiation. To further improve bactericidal activity and endow Ti with immunomodulatory properties, the BaTiO_3−x_ surface was covalently immobilized with L-arginine (BaTiO_3−x_/LA), which has therapeutic potential for enhancing endogenous NO regulation [88]. On the one hand, ultrasound can stimulate the decomposition of L-arginine to produce nitric oxide (NO), initiate the chain reaction of ROS-NO-ONOO- to produce peroxynitrite anion (ONOO^−^), resulting in a higher antibacterial rate of 97.08% against *MRSA*. On the other hand, NO molecules effectively induced the M1 polarization of macrophages to promote phagocytosis in the early stage of bacterial infection. At the same time, the nanorod arrays facilitated the macrophage polarization towards M2 type after bacterial infection was successfully eradicated.

Doping metal elements such as Cu, Zn, and Ag not only leverages their intrinsic antibacterial activities to enhance the bactericidal efficiency, while simultaneously regulating osteogenesis, angiogenesis, and immune responses [89]. For instance, core–shell BTO@MSN-Cu nanocomposites and chitosan were co-deposited onto Ti substrates via the electrodeposition method [90]. The results showed that ROS levels generated by BaTiO_3_ (BTO) core under ultrasound excitation were insufficient to kill bacteria (65.9% inhibition rate for *S. aureus* and 45.6% inhibition rate for *E. coli*). When combined with released copper ions, the surface achieved high bactericidal efficiency against *S. aureus* (>99%) and *E. coli* (>95%). Furthermore, released copper ions can stimulate angiogenesis through hypoxia-inducible factor 1-alpha (HIF-1α) mediated signaling pathways to promote bone regeneration. Unlike the bacterial death caused by excessive copper accumulation and membrane disruption, recent studies have identified a new programmed cuproptosis-like death that occurs in bacteria [91]. Generally, abnormally accumulated copper ions might play a role in the aggregation of thioctanoylated proteins, which inhibits the TCA cycle and suppresses the electron transport chain enzyme activity, resulting in metabolic dysregulation and bacterial cell death. Li and co-workers fabricated an ultrasound-triggered cuproptosis-like surface on Ti by sequentially coating it with hypoxia-triggered antibiotic tinidazole (TNZ), tinidazole-doped probiotic-derived membrane vesicles (OMVs), and metal–organic sonosensitizer copper-tetrakis (4-carboxyphenyl) porphyrin (Cu-TCPP), which is targeted for bacteria in a hypoxic microenvironment [92]. The results showed that ultrasound-triggered Cu-TCPP could produce ^1^O_2_ under normoxic conditions, which consumed oxygen and potentiated the hypoxic condition, thereby activating TNZ to eradicate the remaining bacteria inside the biofilm. The combined bactericidal ability of Cu-TCPP and TNZ under ultrasound exposure exhibited high antibacterial rates of 99.72% for *S. aureus* and 99.55% for *E. coli*. This study provided a promising solution to combat bacterial infections under hypoxic conditions. 

Constructing heterojunctions is also an important strategy to enhance ultrasound-induced charge transfer and separation, as well as the ultrasound-to-thermal conversion efficiency. Guan et al. developed a TiO_2_/Ti_2_O_3_/vertical graphene metainterface heterostructure film (HTO/VG) based on the metastructure TiO_2_ porous coating reported in their previous study [93]. In this work, they found that the Ti_2_O_3_/VG metainterface accumulated electrons and provided reaction sites for the catalytic process under ultrasound irradiation (1.2 W/cm^2^, 1.0 MHz, 50% duty cycle, 10 min). Simultaneously, the built-in electric field at the Ti_2_O_3_/TiO_2_ heterointerface (HTO) further drove carrier transfer and greatly promoted the sonodynamic effects. Combined with the sonothermal effects of in situ-grown “honeycomb-like” micrometer-scale superstructures, HTO/VG exhibited an impressive antibacterial rate of 98.22% against *S. aureus* and good biofilm eradication.

As discussed above, combination therapy is more effective at destroying bacteria and eradicating biofilm than single therapy, and it can also broaden the bactericidal spectrum. However, incorporating multiple components or exposing them to multiple stimuli raises biocompatibility and safety concerns. Therefore, it is imperative to explore ultrasound-responsive surfaces composed of nutrient elements in the human body, such as calcium (Ca), phosphorus (P), magnesium (Mg), and silicon (Si).

**Table 2:** Multimodal antibacterial surfaces on Titanium alloys.

Multimodal Surfaces	Antibacterial Efficiency	Condition	Ref.
OVs-enriched PDA/TiCu (SDT + PDT + PTT)	99.19% (*E. coli*) 95.03% (*S. aureus*)	US: 1.5 W/cm^2^, 5 min NIR-I: 1.5 W/cm^2^, 10 min	[77]
Ti/PDA/BP surface (SDT + PTT)	96.6% (*S. aureus*)	US: 1.5 W/cm^2^, 20 min NIR-I: 1.0 W/cm^2^, 10 min	[85]
Ti-S-TiO_2−x_ (SDT + PTT)	99.995% (*S. aureus*)	US: 1.5 W/cm^2^, 10 min NIR-I: 0.35 W/cm^2^, 5 min	[86]
BaTiO_3−x_/LA nanoarray (SDT + immunotherapy)	97.08% (*MRSA*)	US: 1.5 W/cm^2^, 15 min	[87]
Ti/pTA/TNZ/Cu-TOT (SDT + chemotherapy)	99.55% (*E. coli*) 99.72% (*S. aureus*)	US: 1.5 W/cm^2^, 10 min	[92]
TiO_2_/Ti_2_O_3_/vertical graphene (SDT + STT)	99.54% (*E. coli*) 99.99% (*S. aureus*)	US: 1.2 W/cm^2^, 10 min	[93]
F-doped TiO_2_ surface (SDT + NIR)	99.7% (*S. aureus* film)	US: 1.5 W/cm^2^, 15 min NIR-II: 1.0 W/cm^2^, 15 min	[94]

## 5. Conclusions and Future Perspectives

To date, the design and construction of ultrasound-responsive surfaces or coatings on orthopedic implants have emerged as an innovative tool for treating implant-associated bacterial infection. These surfaces and coatings have unique traits that allow them to activate antibacterial therapy in cases of surgical site infection. In clinical practice, systemic and local antibiotic administration remains the predominant treatment to eradicate the peri-implant biofilm. However, inadequate antibiotic penetration into the biofilm matrix undermines its therapeutic effectiveness. Secondary surgery is then required to remove the biofilm through mechanical debridement or replace the infected implants. Compared with these surgical interventions, ultrasound is a minimally invasive or non-invasive modality that avoids revision surgery and its related physical and financial burdens. More importantly, ultrasound can penetrate and disrupt the biofilm extracellular matrix through cavitation. This provides a significant advantage in eradicating bacteria via ROS or thermal effects. Recently, a wide range of antibacterial strategies have also been explored and developed, such as photo-responsive antibacterial coatings, controlled-release antimicrobial coatings, and immune-activating coatings. While these strategies show strong potential to reduce early-stage bacterial colonization, their translational efficiency is profoundly limited by shallow light penetration, a narrow dosage range, mature biofilm, and persistent bacterial cells. By contrast, ultrasound-responsive antibacterial therapies offer deep tissue penetration and spatiotemporal control, and the high concentrations of ROS and thermal effects they generate are transient, effectively minimizing damage to surrounding tissues.

Despite the advancements achieved through these endeavors, there are still numerous challenges in clinical translation. First, safety concerns are the main barrier to clinical translation. The primary one is whether local accumulation of bacterial debris after ultrasound treatment may trigger chronic inflammation and compromise implant osseointegration. Previously cited in vivo immunological and osseointegration studies using infected bone defect models showed that ultrasound-responsive antibacterial surfaces did not provoke notable inflammatory reactions. They also retained strong osteogenic activities to promote new bone formation. This may be ascribed to the bioactive components engineered and incorporated to promote osteogenesis, modulate immune response, and enhance angiogenesis. Nonetheless, this is insufficient to assess safety for clinical translation, and comprehensive evaluations of long-term biosafety and immune responses should be conducted using large animal models. Another safety concern is potential toxicity from nanomaterials and functional elements, such as aluminum (Al), copper (Cu), and gold (Au) incorporated into surfaces or coatings as key components for SDT or STT. Ultrasound irradiation can accelerate the release of embedded nanomaterials and metal ions, which may adversely affect mammalian-cell compatibility, hemocompatibility, and biosafety. These are decisive factors for obtaining approval of medical devices. Second, the long-term stability of surfaces may affect antibacterial efficiency for clinical outcomes. For instance, the release of key components in a physiological environment can reduce sonocatalytic activity after prolonged implantation. Another example is that oxygen vacancies play a crucial role in improving sonocatalytic performance. However, maintaining long-term stability in ambient air is difficult. Because medical implants undergo a long timeline from manufacturing to clinical use, ensuring sonocatalytic properties during storage is a critical problem. Third, several practical considerations must be addressed to accelerate clinical translation of ultrasound-responsive surfaces. The time and number of treatments are the primary concerns and should be tailored according to infection severity and type, as this critically affects the therapeutic efficiency. For instance, preventing early bacterial adhesion and treating established biofilms require distinct ultrasound parameters and treatments. Real-time temperature monitoring is also essential to keep local temperature below 41 and avoid thermal damage, since high-temperature exposure may induce tissue necrosis and inflammatory responses. Additionally, despite the promising antibacterial effects observed in in vivo bone defect models, acoustic accessibility to deep-seated implants remains challenging because tissue attenuation may reduce the effective energy reaching the implant surface.

Moreover, it should be noted that the antibacterial percentages reported across the included studies were obtained under different experimental conditions, including variations in ultrasound equipment, parameters, exposure time, bacterial strain, and assay protocol (Table 1 and Table 2). Thus, our discussion emphasizes the quantitative trends and mechanistic principles rather than absolute values. However, this raises a challenge in directly comparing antibacterial efficiency among materials. Therefore, there is an urgent need to establish a standardized protocol for evaluating antibacterial activity in ultrasound-responsive antibacterial therapies, as well as standardized reporting with detailed parameter specifications. This can provide a foundation for future clinical translation.

As presented and discussed in the review, a variety of promising strategies have been developed to enhance the sonodynamic and sonothermal activities, such as integrating piezoelectric nanomaterials onto surfaces, engineering interfacial defects or heterojunctions, and designing novel metamaterial structures to manipulate acoustic energy. These approaches may pose challenges for acoustic energy harvesting, electron-hole pair separation, and recombination suppression in constructing ultrasound-responsive antibacterial surfaces.

## Data Availability

This review article does not consist of any new data. All data discussed in this review are available from the cited publications.

## Abbreviations

The following abbreviations are used in this manuscript:

IAIs	Implant-associated infection
Ti	Titanium
TiO_2_	Titanium dioxide
US	Ultrasound
ROS	Reactive oxygen species
SDT	Sonodynamic therapy
STT	Sonothermal therapy
PTT	Photothermal therapy
PDT	Photodynamic therapy
OVs	Oxygen vacancies
BTO	Barium titanate (BaTiO_3_)
RP	Red phosphorus
NO	Nitric oxide
QDs	Quantum dots

## Appendix A. Methods

The Web of Science Core Collection Database was searched using the terms ultrasound titanium*, ultrasound antibacterial*, ultrasound infection*, ultrasound coating*, ultrasound surface*, titanium antibacterial*, titanium infection* from 2020 to August 2026. The search included English-language articles that studied antibacterial activity or biofilm eradication through the design and construction of ultrasound-responsive surfaces or coatings on titanium-based implants. Accordingly, studies that used only nanomaterials or hydrogels to combat bacterial infections have not been included. Studies that used therapeutic stimuli other than ultrasound were also excluded. In addition, studies focusing on antibacterial therapies for anatomical locations other than orthopedic or dental tissues were also excluded.
